# The Radiological and Histological Phenotype of Skeletal Abnormalities in Fetal *ARCN1*-Related Syndrome

**DOI:** 10.1177/10935266231213785

**Published:** 2023-12-03

**Authors:** Charlotte A. Houck, Marije Koopmans, Peter G. J. Nikkels

**Affiliations:** 1 Department of Pathology, University Medical Center Utrecht, Utrecht, The Netherlands; 2 Department of Clinical Genetics, University Medical Center Utrecht, Utrecht, The Netherlands

**Keywords:** autopsy, fetal, congenital anomaly, skeletal dysplasia

## Abstract

Mutations in *ARCN1* give rise to a syndromic disorder with rhizomelic short stature with microretrognathia and developmental delay. *ARCN1* encodes the delta subunit of the coat protein I complex, which is required for intracellular trafficking of collagen 1 and which may also be involved in the endoplasmic reticulum (ER) stress response. In this paper we describe for the first time the skeletal histological abnormalities in an 18-week-old fetus with an *ARCN1* mutation, and we suggest that the skeletal phenotype in *ARCN1*-related syndrome has more resemblance with ER stress than with a defect in collagen 1 metabolism.

## Introduction

The phenotypic spectrum of *ARCN1*-related syndrome has been described in 15 patients and 5 fetal cases in the past 6 years.^1-4^ Core features include fetal growth restriction and micrognathia, and other common features are genitourinary anomalies, microcephaly and developmental delay. In contrast to liveborn cases, fetal cases more often have rhizomelic shortening and skeletal anomalies.^4^

*ARCN1* encodes the coatomer subunit delta of coat protein complex I (COPI), which is essential for intracellular transport of type 1 collagen.^1^ Another example of deficient COPI transport of collagen is due to mutations in KDELR2 and shows a severe osteogenesis imperfecta (OI) phenotype.^5^ Furthermore, Izumi et al^1^ showed that mutations in *ARCN1* not only impair intracellular collagen transport, but also trigger an endoplasmic reticulum (ER) stress response. Loss-of-function mutations in *ARCN1* result in a reduction of collagen secretion, which is thought to represent the mechanism underlying the skeletal phenotype of *ARCN1*-related syndrome. The histological phenotype of this disorder has never been described. We hereby present the result of post-mortem examination, including histological examination, of a case of fetal *ARCN1*-related syndrome.

## Case report

A 28-year-old woman (G2P1) with an uneventful medical history was referred to the Department of Obstetrics and Gynecology on 13 + 5 weeks gestation for evaluation of skeletal anomalies established at routine first trimester ultrasonographic examination. The ribs and long bones were short (below the first percentile). As a severe and potentially lethal skeletal dysplasia was highly suspected, chorionic villus sampling was performed to collect material for genetic testing. Exome sequencing using DNA of the fetus and both parents (“trio-whole exome sequencing”) revealed the following de novo, heterozygous variant in the *ARCN1* gene: Chr11(GRCh37):g.118452226T>C NM_001655.4:c.267+2T>C r.spl. This is a variant in the canonical donor splice site of intron 2 in the *ARCN1* gene, which, according to various splice site prediction programs, may lead to nearly complete deletion of the splice donor site. This may potentially lead to exon skipping (open reading frame stays in-frame) and loss of part of the functional domain. This variant has never been reported. It is regarded as “likely pathogenic.” No other mutations were found. Based on the result of genetic testing and the severity of the skeletal anomalies, the patient opted for termination of pregnancy at 18 weeks gestation.

**Figure 1.**
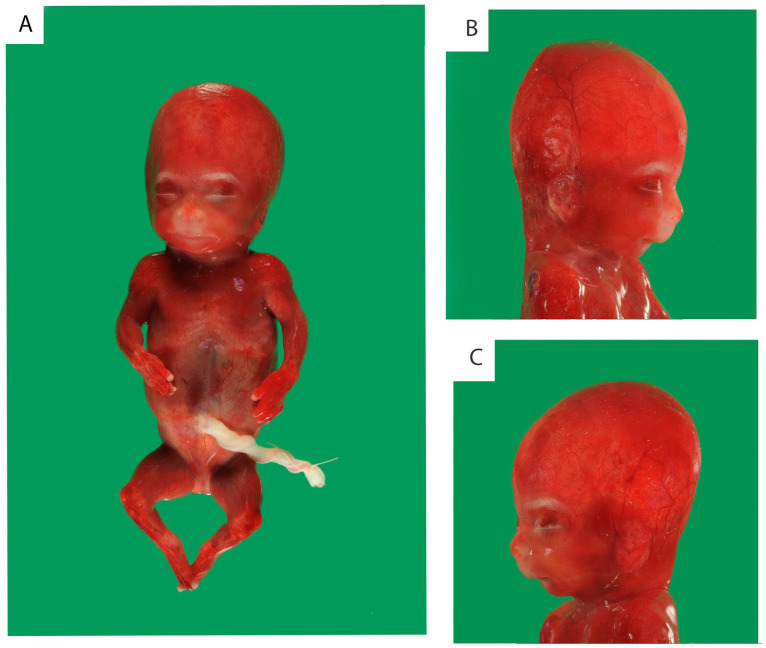
External examination showed signs of fetal growth restriction, micrognathia, short extremities, and mild dysmorphic features (a bulbous nasal tip and a long philtrum).

At autopsy, a female, nonmacerated fetus was seen (Figure 1). In accordance with the previously described fetal phenotype^4^, the fetus showed signs of fetal growth restriction. Body weight (120 g), crown-rump length (12.5 cm), and crown-heel length (16 cm) were according to a gestational age of 15 to 16 weeks. Head circumference was 13.5 cm, which was according to a gestational age of 17 weeks. Furthermore, the fetus had micrognathia, short extremities, and mild dysmorphic features (a bulbous nasal tip and a long philtrum).

**Figure 2.**
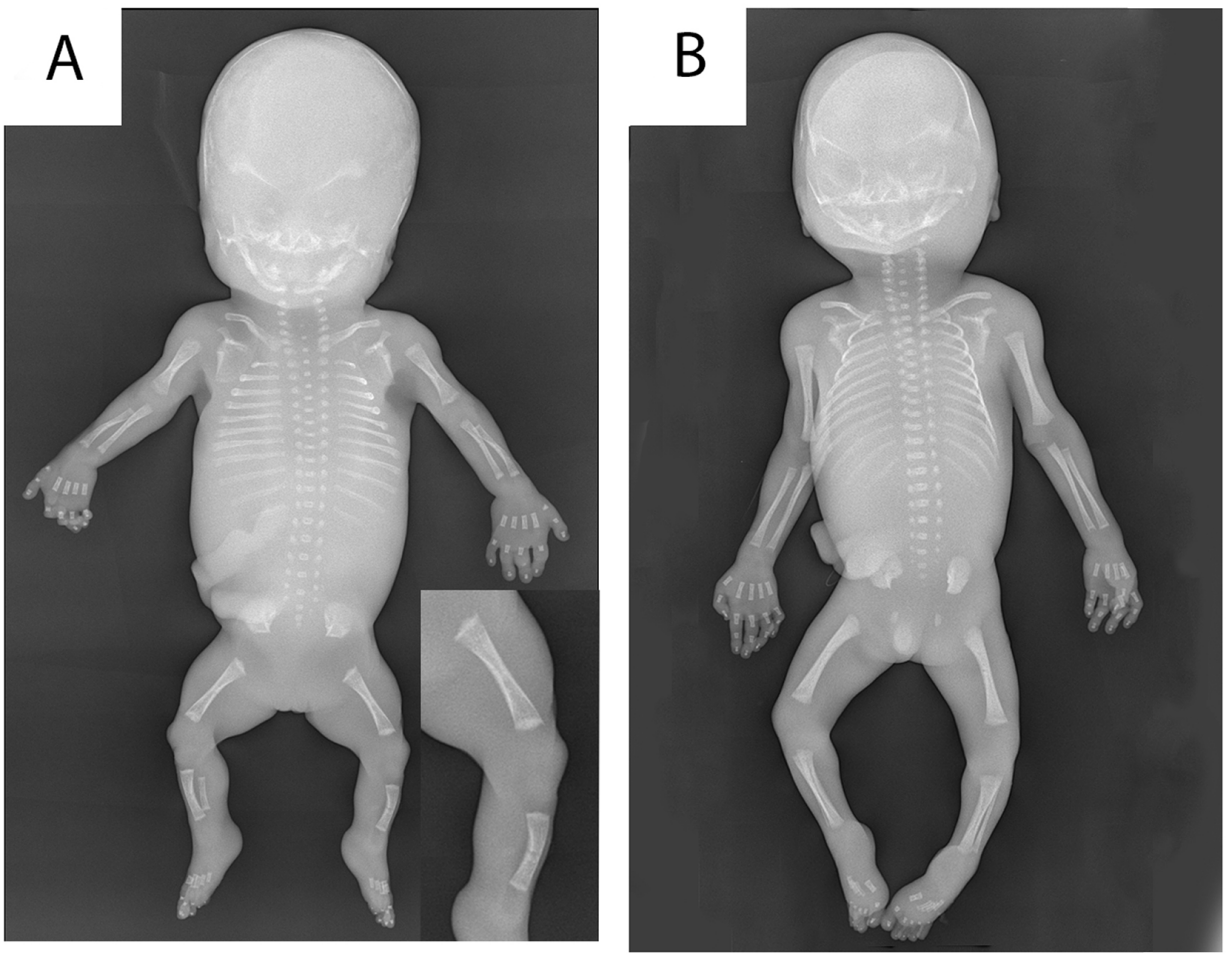
Radiographic examination of our patient with *ARCN1*-related syndrome (A) compared with an age-matched control (B) showed short lengths of long bones, irregular epiphyses (inset), short ribs, platyspondyly, and slightly reduced ossification of the diaphyses, skull, vertebral bodies, and pubic bones.

Post-mortem radiographic examination showed short long bones and ribs (Figure 2). Length of tibia and fibula was normal for a gestational age of 13 weeks, of humerus and femur normal for 14 weeks, and of radius and ulna normal for 15 weeks.^6^ The epiphyses were irregular and the diaphyses showed slightly reduced ossification. Additional findings included platyspondyly (mainly of thoracic vertebral bodies) and slightly reduced ossification of vertebral bodies and pubic bones. There were 11 pairs of ribs. The skull showed a minimally reduced ossification.

Internal examination revealed a normal anatomy. All organ weights were according to a gestational age of 14 to 16 weeks.^7^ The lungs weighed 3.2 g, which was 2.67% of the body weight (low-normal for gestational age; 3.14 ± 0.84).^8^

**Figure 3.**
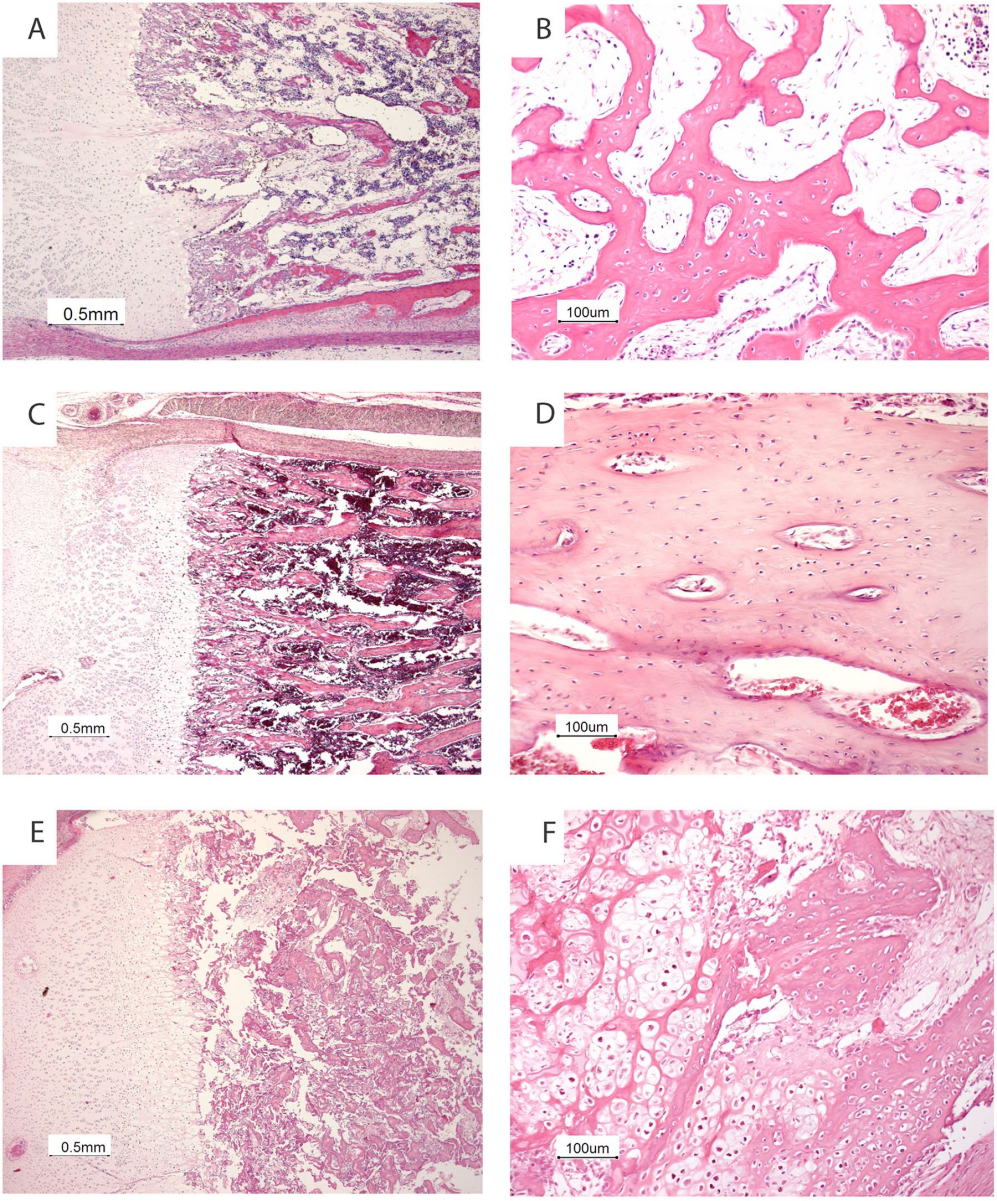
Skeletal histology of our patient with *ARCN1*-related syndrome (A, B) compared with an age matched control (C, D) and with a fetus with a *KDELR2* mutation (E, F) shows a relatively thin hypertrophic zone (A), an irregular zone of chondro-osseous transformation with persisting islets of cartilage matrix and chondrocytes beyond the growth plate (A) and relatively cellular bony trabeculae (B). The histology of bone and cartilage from the fetus with the *KDELR2* mutation shows the typical histology of severe osteogenesis imperfecta with a normal growth plate (E) and hypercellular bony trabeculae and metaplastic cartilage at the site of a microfracture (F).

Bone specimens (rib, femur, and tibia) were decalcified using an EDTA solution. Figure 3 shows the histological examination in our patient compared with an age matched control and with histology from a fetus with a *KDELR2* mutation with typical histology from a severe osteogenesis imperfecta phenotype. In our *ARCN1-*patient, the growth plate showed a relatively thin hypertrophic zone, and the proliferative zone had a normal width. The zone of chondro-osseous transformation was irregular. Foci of not only persisting cartilage matrix but also chondrocytes were observed within the bony trabeculae of the primary spongiosa. Furthermore, the bony trabeculae were relatively cellular and consisted mainly of woven bone. Histological sections of the lungs showed a predominantly pseudoglandular pattern, indicating a slightly delayed maturation for the gestational age of 18 weeks.

## Discussion

It was recently shown that loss-of-function mutations in *ARCN1* cause defective COPI-mediated intracellular transport of type 1 collagen, thereby leading to reduced type 1 collagen secretion.^1^ It was suggested that this may be the cause of skeletal abnormalities in patients with these mutations. Skeletal abnormalities were present in all previously reported cases with *ARCN1-*related syndrome,^4^ as well as in our case.

Defects in type 1 collagen metabolism in relation to skeletal abnormalities have been extensively described in the context of OI. An estimated 90% of cases with OI is due to mutations in type 1 collagen genes (*COL1A1* or *COL1A2*), leading to a reduced amount or complete absence of normal type 1 collagen.^9^ Several other types of OI are caused by mutations in genes involved in intracellular trafficking. For instance, mutations in *KDELR2*, a gene involved in retrograde COPI transport, were shown to cause OI.^5^ Despite the mechanistic similarities between this specific form of OI and *ARCN1*-related syndrome, radiological and histological findings are markedly different. Patients with mutations in *KDELR2* displayed a typical severe OI phenotype including bone fractures and the histologic presence of metaplastic cartilage in the metaphysis, without any abnormalities in the epiphysis (Figure 3 and personal observations P.G.J.N.). However, radiological examination in our case showed several characteristics which are unusual for OI, including severe growth restriction, absence of bone fractures, irregular epiphyses, and only minimally reduced ossification of the skull. Furthermore, histological abnormalities in our case were mainly seen in the epiphysis. The only—albeit subtle—histological similarity to OI in our case consisted of the relatively cellular bony trabeculae.

Given the discrepancy in radiological and histological findings between *ARCN1*-related syndrome and OI, it could be hypothesized that the skeletal abnormalities in *ARCN1*-related syndrome may (at least in part) be caused by a different mechanism. Izumi et al^1^ showed that mutations in *ARCN1* not only impair intracellular collagen transport, but also trigger an ER stress response, as a result of accumulation of protein by disruption of intracellular trafficking. Previous studies demonstrated that ER stress in chondrocytes may contribute to reduced bone growth.^10-12^ ER stress has been shown to influence essential processes of endochondral ossification, including extracellular matrix protein synthesis, chondrocyte proliferation, and differentiation and regulation of autophagy and apoptosis, resulting in a chondrodysplasia phenotype that will have the most pronounced effects on the epiphyseal growth plate with reduced bone length as result.^12^ Kung et al^10^ demonstrated that ER stress due to mutations in cartilage oligomeric matrix protein or matrilin-3 is involved in the disease mechanism of multiple epiphyseal dysplasia (MED) and pseudoachondroplasia (PSACH), 2 types of chondrodysplasias characterized by a short stature. Similar to our case with *ARCN1*-related syndrome, radiographic abnormalities in these disorders are typically located in the epiphyses. To our knowledge, histological characteristics of the long bones in patients with cartilage oligomeric matrix protein or matrilin-3 related PSACH or MED are unknown. Moreover, the consequences of ER stress in general on bone and cartilage have not been described for human cases. In several mouse models, a disrupted ER homeostasis in chondrocytes was shown to lead to the accumulation of poorly folded or misfolded proteins with a similar radiological phenotype and it was suggested that ER stress itself results to the skeletal dysplasia.^12^ In a COMP mutation mouse model it was described that ER stress results in a disturbed articular cartilage structure.^13^ All these disturbed features are expected to have the most profound effect on the epiphyseal growth plate and the costo-chondral junction and might explain the observed histological abnormalities in our case. In this report, we present the result of post-mortem examination in a case of fetal *ARCN1*-related syndrome. Similar to previously described cases, we found severe fetal growth restriction, micrognathia, and mild dysmorphic features. Radiographic examination showed severe growth restriction and irregular epiphyses. Histologic examination of the long bones was characterized by a relatively thin hypertrophic zone, an irregular zone of chondro-osseous transformation with persistence of cartilage matrix and chondrocytes, and relatively hypercellular bony trabeculae. As previously demonstrated, mutations in *ARCN1* cause reduced type 1 collagen secretion and ER stress, which may both be involved in the development of skeletal abnormalities in *ARCN1*-related syndrome. However, radiological and histological characteristics of skeletal abnormalities in our case showed more resemblance to skeletal abnormalities due to ER stress, rather than type 1 collagen deficiency.
